# Squats in Surveys: Investigating the Feasibility of, Compliance With, and Respondents' Performance on Fitness Tasks in Self-Administered Smartphone Surveys Using Acceleration Data

**DOI:** 10.3389/fpubh.2021.627509

**Published:** 2021-09-20

**Authors:** Anne Elevelt, Jan Karem Höhne, Annelies G. Blom

**Affiliations:** ^1^Department of Methodology and Statistics, Utrecht University, Netherlands; ^2^Statistics Netherlands, Netherlands; ^3^Collaborative Research Center 884 “Political Economy of Reforms”, University of Mannheim, Mannheim, Germany; ^4^Research and Expertise Centre for Survey Methodology (RECSM)-Univeristat Pompeu Fabra, Barcelona, Spain; ^5^Data Science, School of Social Sciences, Department of Political Science, University of Mannheim, Mannheim, Germany

**Keywords:** acceleration data, compliance, fitness task, smartphone survey, physical fitness measures, SurveyMotion

## Abstract

Digital health data that accompany data from traditional surveys are becoming increasingly important in health-related research. For instance, smartphones have many built-in sensors, such as accelerometers that measure acceleration so that they offer many new research possibilities. Such acceleration data can be used as a more objective supplement to health and physical fitness measures (or survey questions). In this study, we therefore investigate respondents' compliance with and performance on fitness tasks in self-administered smartphone surveys. For this purpose, we use data from a cross-sectional study as well as a lab study in which we asked respondents to do squats (knee bends). We also employed a variety of questions on respondents' health and fitness level and additionally collected high-frequency acceleration data. Our results reveal that observed compliance is higher than hypothetical compliance. Respondents gave mainly health-related reasons for non-compliance. Respondents' health status positively affects compliance propensities. Finally, the results show that acceleration data of smartphones can be used to validate the compliance with and performance on fitness tasks. These findings indicate that asking respondents to conduct fitness tasks in self-administered smartphone surveys is a feasible endeavor for collecting more objective data on physical fitness levels.

## Introduction and Background

People's physical fitness level is crucial information in medicine and health-related research (1, 2). When it comes to measuring physical fitness, most researchers rely on self-report questions employed in surveys [see International Physical Activity Questionnaire (IPAQ), 36-item Short Form Health Survey (SF-36), or LASA Physical Activity Questionnaire (LAPAQ)]. For instance, the Health and Retirement Study (3) asks respondents the following question: “*Would you say your health is excellent, very good, good, fair, or poor?”* Such self-report questions are subject to respondents' own interpretation and evaluation of their physical fitness (4, 5). In addition, Prince et al. (6) suggest that self-report questions on physical fitness are prone to systematic measurement errors caused by social desirability (e.g., resulting in overreporting) or inaccurate recall (e.g., resulting in over- or underreporting). These methodological problems associated with subjective physical fitness measures in surveys exhibit the potential importance of more objective measures.

Replacing self-report questions with more objective measures on respondents' physical fitness level may decrease systematic measurement errors. Therefore, large-scale national and international health-related surveys, such as the Health and Retirement Study (HRS), the Survey of Health, Aging and Retirement in Europe (SHARE), and the English Longitudinal Study on Aging (ELSA), have regularly employed additional tasks to objectively measure respondents' physical fitness. In a pilot in 2006, the HRS, for instance, added several physical fitness tasks, such as a balance test (i.e., asking respondents to stand for 10 s at a fixed point without stepping away from it) and a walking test (i.e., asking respondents to walk about 2 m in a straight line), to its core survey modules. These tasks were overseen by and conducted with an interviewer present during the interview. Sakshaug et al. (7) reported that about 93% of the eligible HRS respondents complied with these fitness tasks. This high compliance rate might be due to the interviewer-administered survey setting. The presence of an interviewer may encourage respondents to participate in fitness tasks; a luxury not available in self-administered survey settings, such as web survey settings (8).

Recently, many major interviewer-administered surveys, including major health-related surveys, switch to or experiment with self-administered web survey settings to be more cost and time efficient. For instance, since 2003 the HRS has assigned sub-samples of their respondents to participate in self-administered web surveys in an attempt to extend their ways of data collection.

This trend toward web survey settings opens novel ways to collect additional data that complement survey responses (9). This especially applies to mobile web surveys that are completed with mobile devices, such as smartphones (9–15). Smartphone use in web surveys is rapidly increasing (16, 17). From a measurement perspective, smartphones are attractive because they contain a variety of built-in sensors, such as accelerometers that measure acceleration, which is defined as the rate of change of velocity of an object over time. Acceleration data provide information about respondents' physiological states, such as movements, allowing researchers to infer respondents' completion conditions in surveys.

There is an increasing number of studies evaluating the usefulness and usability of acceleration data in smartphone surveys (18–20). For instance, Höhne et al. (19) investigated respondents' compliance with simple motion tasks, such as standing at a fixed point (as in a balance test) and walking around (as in a walking test), in a self-administered smartphone survey using acceleration data. The authors found compliance rates of about 90%, which correspond to the compliance rate of the interviewer-administered HRS 2006 pilot [see (7)]. In addition, the acceleration data of smartphones provided supporting evidence for respondents' compliance with the motion tasks.

The results from Höhne et al. (19) indicate the general feasibility of fitness tasks in self-administered smartphone surveys to collect more objective measures of respondents' physical fitness. They also indicate that acceleration data of smartphones can be used to validate respondents' compliance with fitness tasks without requiring the presence of interviewers that oversee their completion. However, the small body of research on the compliance with fitness tasks, coupled with the limited number of fitness tasks tested so far, merits further investigation of the feasibility of fitness tasks in self-administered smartphone surveys.

In the present study, we go beyond existing studies and investigate respondents' compliance with doing squats (knee bends) for 1 min. For this purpose, we conducted self-administered smartphone surveys in a field and a lab setting and collected high-frequency acceleration data of respondents' smartphones. Since the collection of the acceleration data occurs passively (in the background) there is no additional burden for respondents other than doing the squats and holding the smartphone during this task.

In what follows, we describe the research questions, the study design and passive data collection, the task instructions and survey questions used, the underlying samples (cross-sectional study and lab study), and the analytical strategies. We then present the results of the study. Finally, we discuss practical implications associated with the feasibility of fitness tasks in self-administered smartphone surveys and address future research perspectives.

## Research Questions

We start by making a distinction between hypothetical and observed compliance. While hypothetical compliance refers to respondents' general disposition to participate in a task, observed compliance, in contrast, refers to respondents' actual participation in a task. Empirical findings indicate that respondents' hypothetical compliance tends to be higher than their observed compliance with a task (21, 22). Following this relation between hypothetical and observed compliance, we address the following research question: *Do hypothetical and observed compliance rates with fitness tasks in a self-administered smartphone survey differ from each other (RQ1)?*

Further, it is important to explore the reasons for non-compliance as these provide insights into respondents' decision process. Understanding respondents' reasons for non-compliance can help overcoming those reasons or encouraging respondents to comply in future studies. For instance, Höhne et al. (19) investigated the reasons for non-compliance with simple motion tasks and found that respondents mainly reported issues related to health, surroundings, and situation. Thus, we address the following research question: *What are possible reasons for non-compliance with fitness tasks in a self-administered smartphone survey (RQ2)?*

Since unequal compliance propensities across key respondent groups may bias the sample it is important to investigate differences between respondents who comply and those who do not (17, 23–26). In the HRS sample, for instance, respondents who complied with the additional fitness tasks (i.e., balance and walking tests) were more likely to be higher educated and had better self-reported health ratings (7). Therefore, we address the following research question: *What respondent characteristics affect compliance with fitness tasks in a self-administered smartphone survey (RQ3)?*

In the HRS 2006 pilot, an interviewer has overseen the balance and walking tests to monitor respondents' compliance. As demonstrated by Höhne et al. (19), however, such simple fitness tasks are also feasible in self-administered smartphone surveys. The authors argue that acceleration data of respondents' smartphones can potentially be used to monitor and validate respondents' compliance with fitness tasks without interviewers. Accordingly, we address the following research question: *Can acceleration data be used to validate compliance with fitness tasks in a self-administered smartphone survey (RQ4)?*

The interviewer presence in the HRS 2006 pilot was not only important to the monitoring of respondents' compliance with the fitness tasks, but also to the monitoring of respondents' actual performance on the tasks. For instance, do respondents accurately perform the requested tasks or do they take shortcuts introducing measurement errors? Rowlands et al. (27) have shown that acceleration data metrics from GENEactive accelerometers can be used as a complementary description of people's activity profile associated with fitness tasks and physical functions. The authors argue that acceleration data from smartphones are a useful source to evaluate respondents' performance on fitness tasks. Thus, we address a final research question: *Can acceleration data be used to validate respondents' performance (i.e., number of squats) on fitness tasks in a lab study (RQ5)?*

## Method

### Data Sources and Study Designs

In this study, we use two different data sources: Data from a cross-sectional study (*data source 1)* and data from a lab study (*data source 2*). Both data sources contain high-frequency acceleration data collected from respondents' smartphones through the open-source JavaScript-based tool “SurveyMotion (SMotion)” developed by Höhne et al. (19). SMotion collects the total acceleration (TA) of mobile devices, such as smartphones, on a survey page or question level, which is defined as follows:


(1) $$\begin{array}{r} TA\;\left(Total\;Acceleration\right)=\sqrt{a_{x}^{2}+\;a_{y}^{2}+\;a_{z}^{2}} \end{array}$$

Equation 1. Determining Total Acceleration (TA).

*Note:* Accelerations (a) along the x-, y-, and z-axis are defined as a_x_, a_y_, and a_z_, respectively. The International System unit for acceleration is meter per second squared (m/s^2^).

In this study, we calculated the average total acceleration for each respondent on the survey page on which respondents were required to do the squats. These average total acceleration values were based on the raw total acceleration data without checking for exceptionally low or high values because these values reflect specific characteristics of different motion levels that need to be preserved.

In general, the total acceleration of smartphones can be measured with and without gravity depending on the type of built-in accelerometer. Some old and/or low-budget devices are not equipped with all sensors necessary for the measurement of pure total acceleration without gravity. Devices capable of measuring acceleration without gravity integrate the information from three different sensors (i.e., accelerometer, gyroscope, and magnetometer) which through appropriate algorithms are capable of subtracting the gravitational acceleration and thus offering the acceleration without gravity. In these cases, only the total acceleration with gravity can be measured. We conducted all analyses using total acceleration data with gravity to keep the dataset as large as possible (19).

The sampling rate of the total acceleration primarily depends on the device and/or on frequency restrictions set in the JavaScript code. In this study, the total acceleration of smartphones was measured without any frequency restrictions set in the JavaScript code to register it as precisely as possible. On average, the total acceleration was measured every 19 ms.

In addition, we collected several types of paradata, such as response times, by using the open-source JavaScript-based tool “Embedded Client Side Paradata (ECSP)” (28). Prior informed consent for the collection of total acceleration data and paradata was obtained by the survey company as part of panelists' registration process (cross-sectional study; *data source 1*). We also obtained informed consent in the lab study (*data source 2*).

The dataset of the cross-sectional study (*data source 1*) serves for investigating respondents' (hypothetical and observed) compliance, reasons for non-compliance, respondent characteristics associated with compliance, and the validation of compliance (using total acceleration data) in a field setting *(RQ1 to 4)*. The dataset of the lab study *(data source 2)* serves for evaluating squat performance; i.e., the number of performed squats counted by the experimenter *(RQ5)*.

#### Data Source 1: Cross-Sectional Study

This cross-sectional study was conducted by the survey company Respondi in Germany in September and October 2018. Respondi drew a quota sample from their opt-in panel based on age, education, and gender, resulting in a 3 × 3 × 2 quota plan. The company invited respondents by email. The email included an invitation to take part in the survey, an instruction to use a smartphone for survey completion, and a URL link that directed respondents to the smartphone survey. Once there, an introductory page informed respondents about the procedure of the survey and that their data would be treated confidentially. Our study was part of a larger survey with several unrelated studies and was located in the last quarter of the survey.

A total of 1,172 respondents participated in the survey. Some respondents were ineligible because they only visited the title page or they broke-off the survey before being asked any study-relevant questions (*n* = 197). In total, *n* = 975 respondents remained for statistical analyses. Another 27 respondents were excluded because there were some technical difficulties with the acquisition of the total acceleration data. Therefore, *n* = 948 respondents remained for the validation of their squat task compliance by using total acceleration data.

These respondents were aged 18–70 years old, with a mean age of 48.0 (SD = 15.2), and 42.5% of them were female. In terms of education, 43.6% had graduated from a lower secondary school (low education level), 25.2% from an intermediate secondary school (middle education level), and 31.2% from a college preparatory secondary school or university (high education level).

#### Data Source 2: Lab Study

In February 2020, we conducted an additional lab study in Utrecht to get reference data on respondents' squat performance using total acceleration. At this lab study, an experimenter observed and validated respondents' task (or squat) performance. Similar to the cross-sectional study (*data source 1*), respondents were asked to perform squats for 1 min, while collecting the total acceleration of their smartphones. The experimenter observed respondents' compliance with the squat task and manually counted the number of squats that respondents performed.

Data were obtained from 10 adult respondents aged 26–63 years, with a mean age of 33.4 (SD = 11.4), and 50.0% of them were female. In terms of education, all respondents graduated from a college preparatory secondary school or university (high education level). All respondents volunteered willingly and were familiar with the overseeing experimenter.

### Survey Questions and Task Instructions

#### Data Source 1: Cross-Sectional Study

We employed 15 questions that dealt with respondents' fitness level (five questions), general health (one question), and physical functioning (nine questions). These questions were adopted from the Short Form (36) Health Survey [SF-36] (29) and from a study by Keith et al. (30). We also asked about respondents' body weight (one question) and body height (one question) to determine their Body Mass Index (BMI). All questions were presented with vertically aligned response scales and radio buttons (see Appendix A for English translations of all questions and response categories).

After the questions on fitness level, general health, physical functioning, and body weight and height, we asked respondents' about their hypothetical compliance with a fitness task during survey participation. More specifically, we asked the following question with “Yes, I can imagine it” and “No, I cannot imagine it” as response categories: “*In general, could you imagine participating in a fitness task during a survey?”*

We then asked respondents to actually do squats for 1 min while holding their smartphone at chest level. To avoid an artificially sounding instruction, we slightly adapted the request for respondents who initially indicated that they would not comply with a fitness task or who did not provide an answer at all. All respondents received the opportunity to refuse their participation in the squat task by providing a reason for non-compliance in an open answer box. Complying respondents were directed to a survey page displaying a timer counting down from 60 to 0 s. Finally, we asked respondents how many squats they did by providing an open answer box to enter the number of squats.

**Figure 1:**
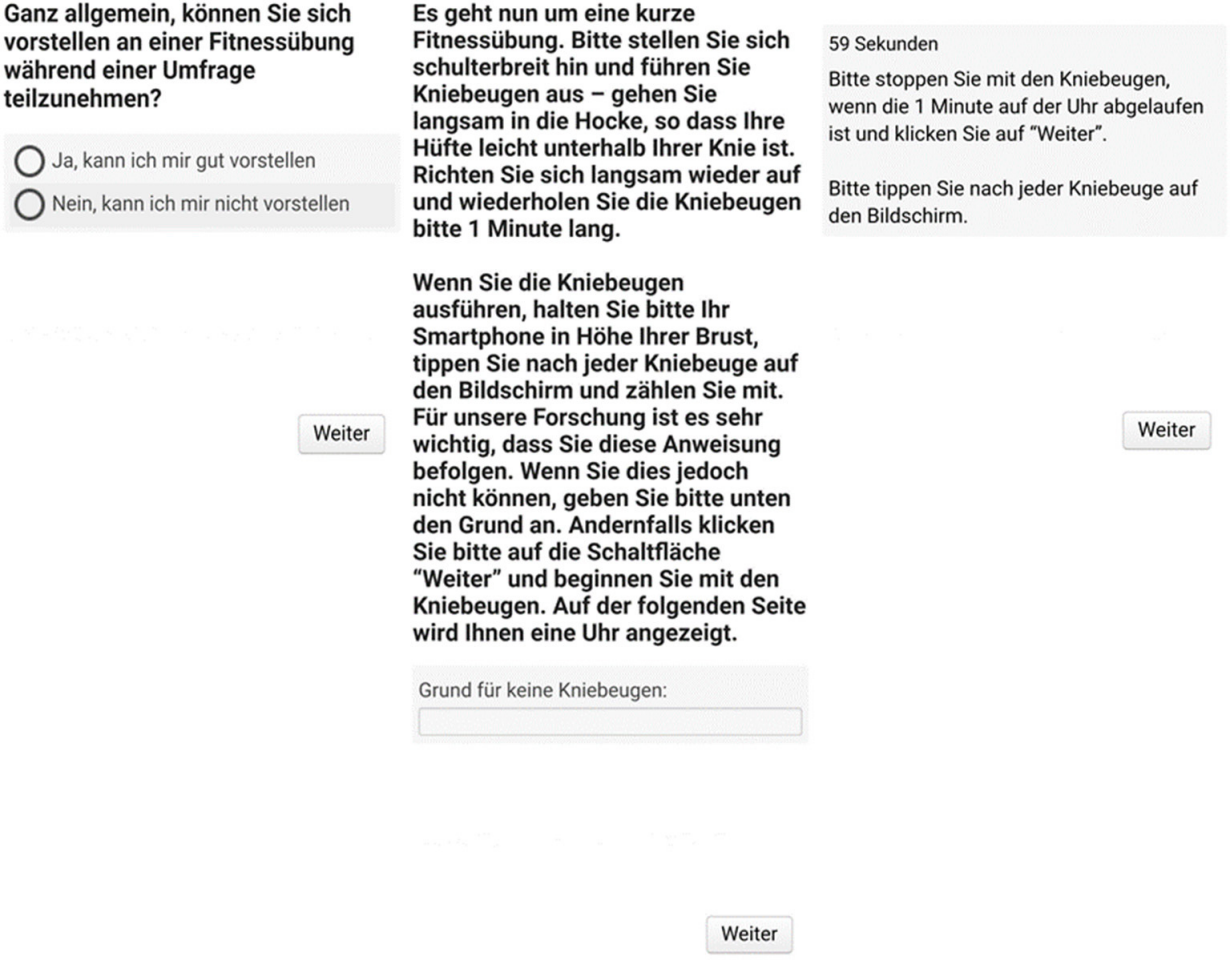
Screenshots for hypothetical compliance (on the left), observed compliance including squat instruction (in the middle), and the timer page for doing squats (on the right). The German versions of all questions and instructions are available from the second author on request.

All questions and instructions were in German, which was the mother tongue of 94.2% of the respondents. To improve survey completion and task performance, we used an optimized survey layout that avoids horizontal scrolling. Figure 1 displays screenshots for hypothetical compliance, observed compliance including squat instruction, and the timer page for doing squats.

#### Data Source 2: Lab Study

Similar to the cross-sectional study (*data source 1*), respondents in the lab study were asked to perform squats for 1 min. The design of the web survey was identical to the one of the cross-sectional study (see Figure 1). One important difference is that, in the lab study, respondents were asked to do the squats in four different ways, varying their intensity. This was done to ensure variation in the quality and number of squats, emulating real-world variation that is caused by respondents' motivation and skills. The four conditions were as follow:

- Deep squats at high pace (high intensity).
- Easy squats at high pace (medium intensity).
- Deep squats at slow pace (medium intensity).
- Easy squats at slow pace (low intensity).

In order to minimize the occurrence of order effects respondents conducted the four different types of squats in a randomized order. In addition, respondents were able to take breaks between each course of squats to ensure physical endurance. Due to some technical difficulties total acceleration data could not be accurately collected for four out of 40 trials, leaving us with 36 trials for statistical analyzes. The lab study contained no additional survey questions for the respondents, except for some socio-demographic questions.

### Analytical Strategy

We use *data source 1* for research questions 1–4 and *data source 2* for research question 5.

***Research Question 1***. To investigate our first research question on the hypothetical and observed compliance of respondents with doing squats for 1 min, we start by determining respondents' hypothetical compliance. For this purpose, we look at the proportion of respondents saying “Yes, I can imagine it” when they were asked whether they can imagine participating in a fitness task. In a next step, we determined respondents' observed compliance by looking at the proportion of respondents that did not enter any reasons in the open answer box for non-compliance when they were asked to do squats for 1 min. In these cases, we assumed that respondents comply with the instructions, keeping in mind that not providing a reason does not constitute strong proof of compliance. In order to test for differences between hypothetical and observed compliance we conducted a chi-squared test.

***Research Question 2***. To investigate our second research question on respondents' reasons for non-compliance we coded respondents' stated reasons for non-compliance. We classified the open responses into six categories following the example of Höhne et al. (19).

Respondents' stated reasons for non-compliance were coded by two coders. To estimate inter-coder reliability about 13% of the reasons were coded by both coders. Then, we computed Cohen's κ to determine the agreement between the two coders. There was excellent agreement with a Cohen's κ = 0.85.

***Research Question 3***. With respect to our third research question on the variables that are associated with respondents' compliance, we conducted a logistic regression with observed compliance (1 = yes) as binary dependent variable.

To our best of knowledge there are (almost) no empirical studies investigating respondents' compliance with fitness tasks in general and squats in particular. Thus, there is little knowledge on what external variables affect respondents' compliance. An exception is Sakshaug et al. (7) who show that respondents' compliance with fitness tasks highly depends on health status. We therefore include the following health-related variables as independent variables: fitness level, general health, physical functioning, and BMI.

We determined respondents' fitness level using five questions asking how they assess their overall fitness level, endurance, sprint speed, strength, and flexibility. These questions were asked with completely verbalized, five-point rating scales running from 1 “Very good” to 5 “Very bad”. For statistical analyses, we recoded the scales of all questions so that they run from 1 “Very bad” to 5 “Very good.” An explanatory factor analysis with a principal factor method and a promax rotation revealed that all five questions load on one factor that we call fitness level. We saved Bartlett factor scores with higher scores indicating a higher fitness level and used these scores in the logistic regression model. The fitness level factor explained 60.8% of the variance with a Cronbach's α = 0.88.

In order to measure respondents' general health, we employed one self-report question that is frequently asked in health-related surveys, such as the 36-item Short Form Health Survey (SF-36) and the HRS (31). More specifically, respondents were asked how they rate their general health with a completely verbalized, five-point rating scale running either from 1 “Excellent” to 5 “Bad,” or from 1 “Bad” to 5 “Excellent” (the question was part of a scale direction experiment). For statistical analyses, we coded the scale so that it runs from 1 “Bad” to 5 “Excellent”.

We determined a physical functioning score following the scoring scheme proposed by the SF-36 developers (32). More specifically, scores for each of the nine questions are transformed into a scale ranging from 0 (limited a lot by health) to 100 (not limited at all by health). Subsequently, we calculated respondents' average score across all nine questions (32). These questions were asked with completely verbalized, three-point rating scales using the following response categories: 1 “Yes, limits me greatly,” 2 “Yes, limits me somewhat,” and 3 “No, limits me not at all.”

Finally, we calculated the BMI based on respondents' body weight (in kilogram; kg) and body height (in meters; m) that they were asked to provide. The two questions used an open answer box for entering the body weight and body height, respectively. The BMI is defined as the body weight divided by the square of the body height. Its system unit is kg/m^2^.

In addition, we included several socio-demographic control variables in the logistic regression model: Female (1 = yes), age (in years), and education with high as reference: low (1 = yes) and middle (1 = yes). For the logistic regression, we calculate and report Average Marginal Effects (AMEs) and transform them to percentages to facilitate interpretation.

***Research Question 4***. To answer our fourth research question on the validation of respondents' compliance using the total acceleration data, we plotted the course of total acceleration of respondents on the survey page with the timer for doing squats for 1 min. In the plots, the x-axis represents the acceleration measurements over time (in milliseconds) and the y-axis represents the total acceleration measured in meter per second squared (m/s^2^). In a next step, we coded the total acceleration plots and divided them into the following three categories: non-compliance, partial compliance, and full compliance. This was done for all respondents who did not provide a reason for non-compliance.

Again, the total acceleration plots were coded by two coders. To estimate inter-coder reliability about 11% of the plots were coded by both coders. Then, we computed Cohen's κ to determine the agreement between the two coders. There was excellent agreement with a Cohen's κ = 0.86.

In addition, we checked respondents' time on the survey page with the timer. Four respondents who were coded as full compliers based on their plots, were subsequently coded as partial compliers because they left the survey page for doing squats before the timer was at zero.

To test for differences in average total acceleration between the categories of respondents (i.e., non-compliance, partial compliance, and full compliance) we conducted a Welch one-way test using the Games-Howell *post-hoc* correction procedure for unequal variances. We used the Welch one-way test and Games-Howell *post-hoc* procedure because the homogeneity of variances assumption was violated [Levene's test: *F*_(2,460)_ = 104.12, *p* < 0.001] and these tests do not require homogeneity of variances.

***Research Question 5***. To answer our final research question on the validation of squat performance (i.e., the number of squats respondents conducted) we correlate the number of squats counted by the experimenter with respondents' average total acceleration while doing squats for 1 min in a lab setting *(data source 2)*. We calculated a Pearson correlation coefficient. This is done to see whether and to what extent the two measures line up. In doing so, we follow Rowlands et al. (27) who have shown that respondents' average total acceleration correlates with their performance on chair stands (a task that is similar to ours).

## Results

### Research Question 1: Hypothetical and Observed Compliance

With respect to hypothetical compliance we found that 57.7% of the respondents could imagine taking part in a fitness task during web survey completion. Interestingly, we found that observed compliance is somewhat higher. Overall, 60.7% of the respondents stated compliance with doing squats for 1 min. The result of a chi-squared test reveals that observed compliance is significantly higher than hypothetical compliance [χ^2^(1) = 102.03, *p* < 0.001]. This finding differs from previous research on hypothetical and observed compliance (21, 22).

### Research Question 2: Reasons for Observed Non-compliance

To answer our second research question, we investigated respondents' stated reasons for non-compliance with the squat task. As shown in Table 1, respondents' stated reasons for non-compliance were largely related to health issues. About 70% of the respondents who did not comply with the squat task reported health-related issues, such as having arthrosis or being injured. Another 11% of respondents reported surrounding issues, such as being in a (public) transportation vehicle or a café. The remaining 20% reported situational issues (about 4%), such as taking care of a child, reported other reasons (about 4%), such as it is too late, reported nonsense (about 5%), such as “Vfygbvh,” or refused their compliance without providing a reason (about 8%).

**Table 1:** Reasons for non-compliance with the squat task.

**Reasons for non-compliance**	**Percentage (frequencies)**
Health issues	68.7 (263)
Surrounding issues	10.7 (41)
Situational issues	3.9 (15)
Other reasons	3.7 (14)
Nonsense	5.0 (19)
Refusals	8.1 (31)

*Because of rounding the percentages do not add up to 100%*.

### Research Question 3: Predicting Observed Compliance

In order to investigate our third research question, which investigates the factors that are associated with squat task compliance, we conducted a logistic regression with observed compliance (1 = yes) as the dependent variable. Table 2 displays the results in the form of Average Marginal Effects (AMEs) and Standard Errors (SEs). Following the pseudo *R*^2^ by Nagelkerke, the explained variance of the logistic regression model is 0.23.

**Table 2:** Logistic regression of observed compliance with the squat task.

**Independent variables**	**AMEs**	**SEs**
Fitness level	−0.56	2.29
General health	8.61^***^	2.33
Physical functioning	0.51^***^	0.00
BMI	−0.89^**^	0.33
Age	−0.11	0.12
Female	−2.80	3.57
**Education with high as reference**
Low	−9.29^*^	4.27
Middle	−4.73	4.91
Nagelkerke's *R*^2^ = 0.227		

* *p < 0.05*,

** *p < 0.01*,

*** *p < 0.001*.

*Dependent variable: Observed compliance. See “Analytical Strategy” for the coding of all variables. AMEs, Average Marginal Effects; SEs, Standard Errors. Intercept is not significant*.

Taking a closer look at Table 2 it can be observed that all health-related variables are significantly associated with observed compliance. The only exception is fitness level, which does not significantly predict observed compliance. Both general health and physical functioning show a positive association with observed compliance implying that respondents with a higher general health or physical functioning have a higher compliance propensity. The probability of complying with the squat task increases about 8.6% when general health increases one level and about 0.5% when physical functioning increases one point. In contrast, BMI shows a negative association implying that respondents with a lower BMI have a higher compliance propensity. The probability of complying with the squat task decreases about 0.9% when BMI increases by one point. Low education is the only socio-demographic variable that is significantly associated with observed compliance. The compliance probability decreases about 9% for low educated respondents (compared to high educated respondents).

### Research Question 4: Validating Compliance With the Squat Task

To answer our fourth research question, we validated respondents' compliance using the total acceleration data. For this purpose, we coded the total acceleration plots of the survey page on which respondents were required to do the squats for 1 min. We only used respondents who complied with the task by not providing a reason for non-compliance when they were asked to do so.

**Figure 2:**
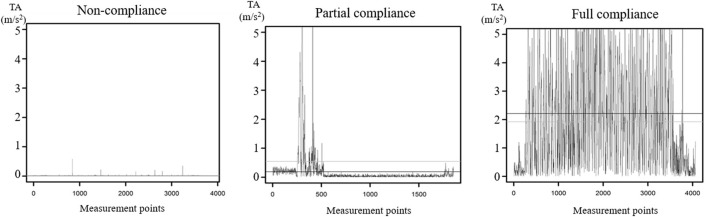
Three example total acceleration plots from three different respondents. While the x-axis represents the total acceleration measurements points (3,600 measurements = 60 s), the y-axis represents the total acceleration measured in meter per second squared (m/s^2^).

Based on their total acceleration plots, we assigned respondents to one out of three compliance categories: Non-compliance, partial compliance, and full compliance. Figure 2 displays example total acceleration plots from three respondents. These plots illustrate the total acceleration of respondents' smartphones while they were required to do squats for 1 min. Total acceleration values lower than 1 indicate no motion [see (18)] and, thus, non-compliance with the squat task. Following this notion, the plot on the left side indicates non-compliance, the plot in the middle indicates partial compliance, and the plot on the right side indicates full compliance.

The results of the coding of the total acceleration plots reveal that the majority of respondents partially (29.5%) or fully complied (42.2%) with the squat task when they agreed to do so. However, there is a substantial minority of respondents who did not comply with the squat task at all (28.3%).

**Figure 3:**
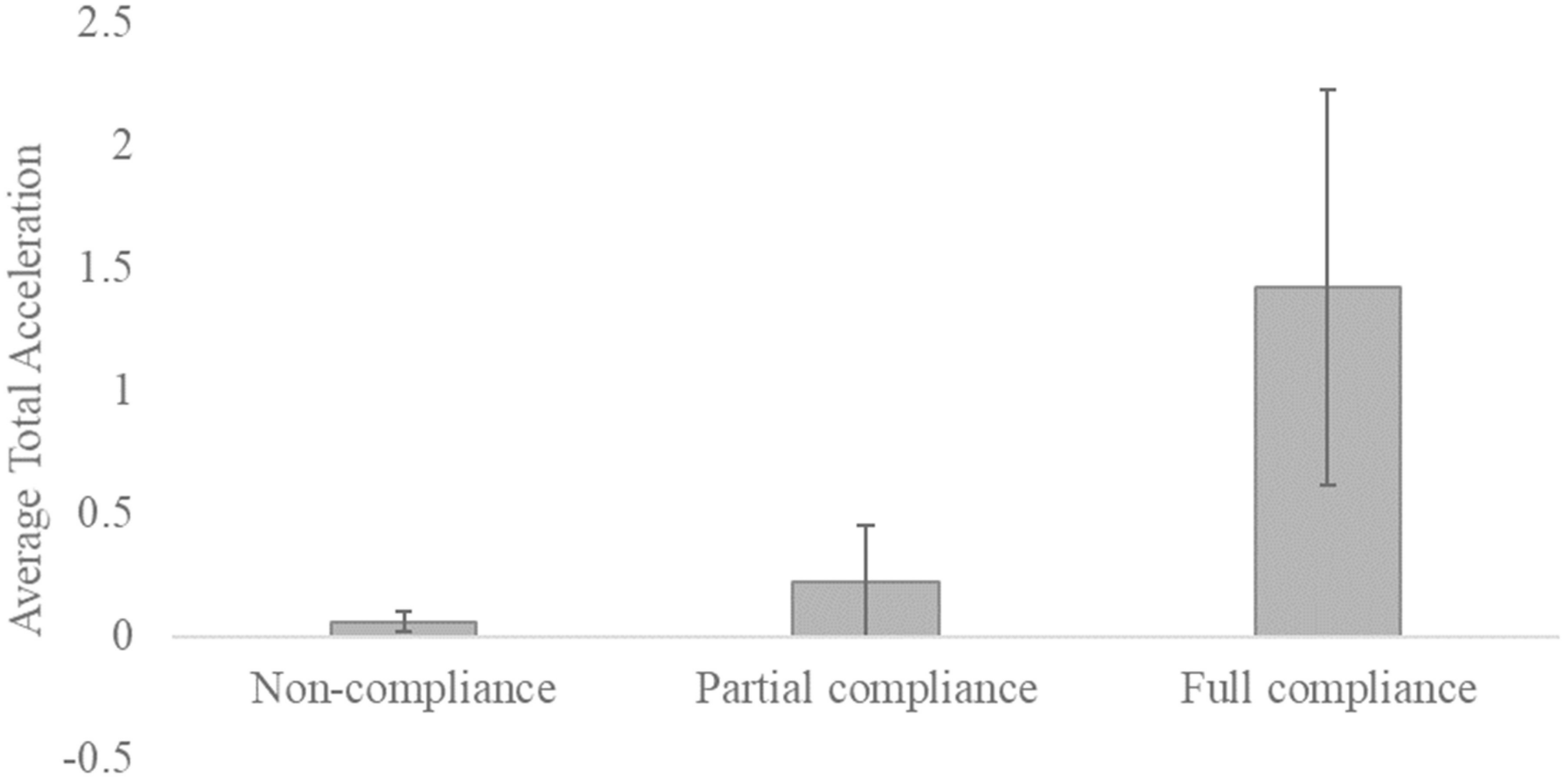
Bar chart of the average total acceleration for the three compliance categories. The vertical lines within the bars represent the standard deviations.

We also tested for total acceleration differences between the compliance categories conducting a Welch one-way test. Figure 3 displays the average total acceleration for the three compliance categories. The results of the Welch one-way test reveal a significant main effect across the three compliance categories [*F*_(2,460)_ = 256.62, *p* < 0.001]. The results of a subsequent *post-hoc* comparison using the Games-Howell procedure indicate significant mean differences between the three compliance categories, except between the non-compliance and the partial compliance categories.

### Research Question 5: Validating Performance of the Squat Task

To answer our final research question on the validation of respondents' squat task performance we now use data from the lab study (*data source 2*). More specifically, we correlate the number of squats counted by the experimenter with respondents' total acceleration data. Pearson's *r* coefficient indicates a high and significant correlation between these two measurements [*r* = 0.77, *p* < 0.001]. This provides supporting evidence that total acceleration data can be used to validate the performance on (or the number of) squats in fitness tasks during self-administered smartphone surveys.

## Discussion

The aim of this study was to investigate the feasibility of fitness tasks in self-administered smartphone surveys. More specifically, we investigated the compliance with and performance on a fitness task asking respondents to do squats for 1 min while collecting high-frequency acceleration data of their smartphones. Our overall findings suggest that such fitness tasks are a feasible endeavor in self-administered smartphone surveys.

With respect to our first research question on differences between hypothetical and observed compliance, we found that observed compliance was significantly higher than hypothetical compliance. This finding differs from findings reported in other studies (21, 22). In our opinion, there are two possible explanations for this phenomenon: First, usually respondents answer survey questions by selecting a response category from a predefined list. This also applies to the smartphone survey in which this study was implemented. Conducting fitness tasks during web surveys is rather seldom and might thus be an interesting and exciting task for respondents. This may lead them to participate, even though they did not intend to do so when asked hypothetically. Future studies may investigate this phenomenon further by asking respondents to outline their motivation for observed compliance. Second, the way of asking (“In general, could you imagine participating in a fitness task during a survey?”) may have affected our results. Respondents may not have perceived this as a real question for hypothetical compliance, but more as an imagination question. In addition, the request for observed compliance was a comparatively intense and guiding question (“If you are not able to do so, please state the reason below”), pushing respondents toward compliance and participation. Future studies could further experiment with different ways of asking for hypothetical and observed compliance in order to optimize compliance questions and increase compliance rates.

Regarding our second research question on potential reasons for non-compliance, we found that the majority of respondents (about 80%) gave reasons related to health, surrounding, or situation. Overall, this finding corresponds to findings reported by Höhne et al. (19), who found that about two thirds of the respondents who did not comply with simple motion tasks reported either health-, surrounding-, or situation-related issues. Note that the comparatively high physical demands of our fitness task may have driven the high prevalence of health-related reasons for non-compliance stated by respondents. Less intensive tasks may cause fewer respondents to refuse compliance with the task because of health-related reasons.

Our third research question dealt with respondents' characteristics that are associated with compliance. In line with previous research, we found that particularly health-related variables affect compliance propensities. Respondents with a lower general health, a lower physical functioning, and a higher BMI are less likely to comply with our fitness task. These respondents may be willing but not able to comply in a squat task. As noted earlier, compliance with less physically demanding tasks than the squat task in our study may result in different correlates of compliance.

With respect to our fourth research question on validating fitness task compliance, we indeed found supporting evidence that acceleration data of smartphones can be used to validate respondents' task compliance in self-administered web surveys. Interestingly, the acceleration data showed that not all respondents who stated compliance (or did not provide a reason for non-compliance) actually complied with doing squats for 1 min. Plotting the course of acceleration data over time reveals that some respondents did not comply at all or only complied partially. Nevertheless, the high observed compliance rate suggests that most respondents comply with a squat task if they agreed to do so. This indicates the general feasibility of fitness tasks in self-administered smartphone surveys to draw conclusions about respondents' physical fitness level.

Finally, regarding our fifth research question on validating the performance on fitness tasks, we found further supporting evidence that acceleration data of smartphones can be used to validate respondents' fitness task performance (i.e., number of squats) in smartphone surveys. This allows us to draw conclusions about the number of squats respondents did. Self-reports of respondents' squat performance probably suffer from an over-reporting of the number of squats due to social desirability. Additionally, using respondents' acceleration data may reduce measurement error. Fitness tasks can thus be used as a more objective supplement to health and physical fitness measures in smartphone surveys.

Our study has some limitations that provide avenues for future research. First, the fitness task was positioned close to the end of the survey. Respondents' compliance might be higher if the task was placed earlier in the survey. Future research could vary the position of the fitness task in the survey (beginning, middle, and end) in order to optimize compliance rates. Second, even though we can validate respondents' performance by counting the number of squats, we cannot make a distinction between good, deep squats, and fast, easy squats yet. Further analyses and more information on the direction of movement could help identifying the quality of the squats. When data on the direction of movement is available for all respondents, performing a peak analysis might be a good way to identify the number of squats. Third, the samples were drawn from an access panel (cross-sectional study) and a volunteer sample (lab study). A probability sample would allow to draw more robust conclusions on fitness task compliance and performance in the general population.

In sum, this study contributes to fitness and health research by proposing a new method to study respondents' physical fitness level. So far, our results indicate that it is feasible to ask respondents to engage in fitness tasks in self-administered smartphone surveys. This increases opportunities for large surveys (e.g., HRS, SHARE, and ELSA) to switch from interviewer-administered surveys to self-administered surveys. We show that compliance with and performance on fitness tasks in self-administered smartphone surveys can be validated with acceleration data. This is much more time- and cost-efficient than employing interviewers and reduces respondent burden because respondents can complete surveys and do fitness tasks without time restrictions. We see a lot of potential for future research employing fitness tasks in self-administered smartphone surveys and extending our task (doing squats) with other commonly used tasks in public health research.

## Data Availability Statement

The raw data supporting the conclusions of this article will be made available by the corresponding author (a.elevelt@cbs.nl), for researchers wishing to verify our results.

## Ethics Statement

Ethical review and approval was not required for the study on human participants in accordance with the local legislation and institutional requirements. The patients/participants provided their written informed consent to participate in this study.

## Author Contributions

AE and JH contributed to the concept and design of the study. JH was responsible for the data collection. AE was responsible for data preparation, analysis, and wrote the first draft of the manuscript. JH and AB critically reviewed the manuscript and wrote some sections of the manuscript. All authors significantly contributed to manuscript and approved the submitted version.

## Conflict of Interest

The authors declare that the research was conducted in the absence of any commercial or financial relationships that could be construed as a potential conflict of interest.

## Publisher's Note

All claims expressed in this article are solely those of the authors and do not necessarily represent those of their affiliated organizations, or those of the publisher, the editors and the reviewers. Any product that may be evaluated in this article, or claim that may be made by its manufacturer, is not guaranteed or endorsed by the publisher.

## APPENDIX A

### Instruction and Question Wording

#### Fitness Level

*Introduction text:* The following questions are about your physical fitness level.

How would you assess your overall fitness level?

How would you assess your endurance?

How would you assess your sprint speed?

How would you assess your strength?

How would you assess your flexibility?

*1 very good – 5 very bad*

*1 very bad – 5 very good*

*Recoded for analysis into 1 very bad – 5 very good*

#### General Health

In general, how would you rate your health?

*1 bad – 5 excellent*

*1 excellent – 5 bad*

*Recoded for analysis into 1 bad – 5 excellent*

#### Physical Functioning

*Introduction text:* Does your health now limit you in the following activities?

Moderate activities, such as moving a table or pushing a vacuum cleaner.

Vigorous activities, such as running or lifting heavy objects.

Lifting or carrying groceries.

Climbing one flight of stairs.

Climbing several flights of stairs.

Bending, kneeling, or stooping.

Walking more than 100 m.

Walking more than a kilometer.

Bathing or dressing yourself.

*1 – yes, limits me greatly. 2 – yes, limits me somewhat. 3 – no, limits me not at all*.

#### BMI

How tall are you?

Please enter your height in meters (m), e.g., 1.76 m.

*Open answer box*.

How much do you weigh?

Please enter your weight in kilogram (kg), e.g., 81.7 kg.

*Open answer box*.

#### Hypothetical Compliance

In general, could you imagine participating in a fitness task during a survey?

*1 yes, I could imagine – 2 no, I could not imagine*

#### Observed Compliance

It is now about a short fitness task. Please stand shoulder wide and perform squats—crouch slowly—so that your hip is slightly below your knees. Straight slowly up and repeat the squats for 1 min.

When doing the squats, hold your phone at chest level, tap the screen after each squat and count the squats you do. For our research it is very important that you follow these instructions. However, if you are not able to do so, please state the reason below. Otherwise, please click on the “Next” button and start with the squats. On the following page, you will see a timer that counts down.

Reason for not being able to do the squats: *[Open answer box:]*

#### Timer Page

*[Timer counting down from 60 to 0 s]*

Please stop with the squats when the 1 min on the timer has expired and click “Next.”

Please tap on the screen after each squat.

#### Number of Squats

How many squats did you do?

Please enter the number of squats:

*Open answer box*.

*Note*. The original German wordings of all questions are available from the second author on request.
